# Utilizing Twins as Controls for Non-Twin Case-Materials in Genome Wide Association Studies

**DOI:** 10.1371/journal.pone.0083101

**Published:** 2013-12-10

**Authors:** Andrea Ganna, Alfredo Ortega-Alonso, Aki Havulinna, Veikko Salomaa, Jaakko Kaprio, Nancy L. Pedersen, Patrick F. Sullivan, Erik Ingelsson, Christina M. Hultman, Patrik K. E. Magnusson

**Affiliations:** 1 Department of Medical Epidemiology and Biostatistics, Karolinska Institutet, Stockholm, Sweden; 2 University of Helsinki, Institute for Molecular Medicine (FIMM), Helsinki, Finland; 3 Department of Chronic Disease Prevention, National Institute for Health and Welfare, Helsinki, Finland; 4 University of Helsinki, Hjelt Institute, Department of Public Health, Helsinki, Finland; 5 National Institute for Health and Welfare, Department of Mental Health and Substance Abuse Services, Helsinki, Finland; 6 FRANZCP, Department of Genetics and Department of Psychiatry, University of North Carolina at Chapel Hill, North Carolina, United States of America; 7 Department of Medical Sciences, Molecular Epidemiology, Uppsala University, Uppsala, Sweden; National Cancer Institute, National Institutes of Health, United States of America

## Abstract

Twin registries around the globe have collected DNA samples from large numbers of monozygotic and dizygotic twins. The twin sample collections are frequently used as controls in disease-specific studies together with non-twins. This approach is unbiased under the hypothesis that twins and singletons are comparable in terms of allele frequencies; i.e. there are no genetic variants associated with being a twin *per se*. To test this hypothesis we performed a genome-wide association study comparing the allele frequency of 572,352 single nucleotide polymorphisms (SNPs) in 1,413 monozygotic (MZ) and 5,451 dizygotic (DZ) twins with 3,720 healthy singletons. Twins and singletons have been genotyped using the same platform. SNPs showing association with being a twin at P-value < 1 × 10^-5^ were selected for replication analysis in 1,492 twins (463 MZ and 1,029 DZ) and 1,880 singletons from Finland. No SNPs reached genome-wide significance (P-value < 5 × 10^-8^) in the main analysis combining MZ and DZ twins. In a secondary analysis including only DZ twins two SNPs (rs2033541 close to *ADAMTSL1* and rs4149283 close to ABCA1) were genome-wide significant after meta-analysis with the Finnish population. The estimated proportion of variance on the liability scale explained by all SNPs was 0.08 (P-value=0.003) when MZ and DZ were considered together and smaller for MZ (0.06, P-value=0.10) compared to DZ (0.09, P-value=0.003) when analyzed separately. In conclusion, twins and singletons can be used in genetic studies together with general population samples without introducing large bias. Further research is needed to explore genetic variances associated with DZ twinning.

## Introduction

Twin brothers and sisters have been extensively studied in Genetic Epidemiology with the aim of disentangling the genetic architecture of multiple biological and behavioral traits. Traditionally, this has been done by mean of quantitative genetic modeling, in which observed and expected variance/covariance structures are estimated and compared utilizing phenotypic twin data [1].

The possibility of using these twin data for such scientific purposes has driven the establishment of multiple large twin cohort studies and registries around the globe [2,3]. 

These registries have traditionally focused on collecting phenotypic data longitudinally on large numbers of participants. However, because of the recent advances in genotyping methods, many twin registries collect DNA from the participating twins. Given the population based nature of most twin cohorts, analyzing this genomic material would allow studying not only the genetic architecture of the collected phenotypes, but also the identical by descent (IBD) sharing of alleles [4], MZ discordant pair analyses [5], gene-environment interactions [6] or joint linkage and association analysis [7]. In addition, the unselected nature of twin participants makes them attractive to be used as controls in disease-specific studies from the same background populations. Nevertheless, in order to develop these approaches in an unbiased manner, twins need to be comparable to singletons in other aspects than the investigated disease. One concern could be that twins have lower birth-weight than singletons [8], mainly due to reduced growth during the third trimester [9]. Low birth-weight has been associated with increased risk of cardiovascular diseases, diabetes and a number of other diseases [10]. Nevertheless health outcomes associated with low birth-weight have been shown to be generally very similar between twins and singletons [11] [12] [13]. A more direct concern for the reliability of the results from genetic association studies using twins as controls is that the genetic architecture of twins and non-twins is comparable and that there are no specific variants associated with being a twin. Previous studies have suggested the existence of a genetic contribution to DZ twinning [14] [15]. However, candidate-gene studies have failed to identify any genetic variance associated with DZ twinning in families [16] [17]. The genetic contribution to MZ twinning has been debated, and although it is generally accepted to be a spontaneous event, uninfluenced by genetic factor, maternal age, parity or race, some studies have suggested that a propensity to MZ twinning can be inherited through the maternal line [18].

We first performed a genome-wide association (GWA) study comparing twins participating in the TwinGene study with healthy singletons enrolled as controls in a large study on schizophrenia susceptibility. Second, we attempted to replicate SNPs showing association with being a twin at P-value < 1 × 10^-5^ in a sample of twins and singletons from Finland. 

Since most twin studies jointly used MZ and DZ in GWAS, we focused on this combined outcome in our primary analysis. In secondary analysis DZ twins were studied separately. This stratified analysis was justified by two reasons. First, some twin materials may consist of purely MZ or DZ twins and second, if genetic variants predisposing to multiple ovulations exist, their detection would be improved by including only DZ twins given the differences in the biological origins of MZ and DZ twins [19].

## Methods

### Study sample

#### TwinGene

The Swedish Twin Registry is a population-based national register currently including close to 200,000 Swedish twins born from 1886 to 2008 [20]. TwinGene is a sub-study that has been conducted within The Swedish Twin Registry to examine associations between genetic factors and common complex disease. Twins born before 1958 were contacted to participate between April 2004 and December 2008. Health and medication data were collected from self-report questionnaires and blood sampling material was mailed to the subject who then contacted a local health care center for blood sampling and a health check-up. In the present analysis we include all the individuals whose co-twin did not participate and randomly selected one individual from each twin pair in which both members participated. This was done to address our research objectives without the added complexity of modeling familial clustering. In total, N=6,886 twins were included in the current study. All the participants in the TwinGene study gave written informed consent and the Ethics Committee of Karolinska Institutet approved the study.

#### Population Controls

The controls from a large study on schizophrenia susceptibility were used as comparison non-twin “control” material [21]. The schizophrenia study was conducted concomitantly with TwinGene and at the same department (Medical Epidemiology and Biostatistics, Karolinska Institutet). The same procedures for collection of blood, extraction of DNA and storage were implemented by the same biobank (KI Biobank) as for TwinGene. The schizophrenia study consists of 11,244 individuals (5,001 cases and 6,243 controls) collected during 6 study waves. The controls had to be born in Sweden or another Nordic country and were identified from national population registers and frequency matched to cases by age, gender and county of residence. Controls had never received a discharge diagnosis of schizophrenia or bipolar disorder. To avoid potential bias introduced from considering schizophrenic patients, we only included the schizophrenia control subjects as controls in our study. Furthermore, due to heterogeneity in genotyping platforms, we only included the fifth and sixth wave of the Swedish schizophrenia study, which used the same platforms as TwinGene (Illumina OmniExpress), giving a total of N=3,729 singleton controls. All the participants in this study gave written informed consent and the Ethics Committee of Karolinska Institutet approved the study.

#### The Finnish twin cohort

Part of the replication analyses utilized data from the FinnTwin12 (FT12) and FinnTwin16 (FT16) cohort studies. Briefly, both the FT12 and FT16 are population based-cohort longitudinal studies including five consecutive birth cohorts of Finnish twins born between 1983 and 1987 (FT12) and between 1975 and 1979 (FT16). Initially, all twins and their parents were approached by letter and invited to participate in the autumn of the year in which their birth cohort reached 11 years of age (FT12), or in the 1-2 months following the twins’ 16th birthday (FT16). The response rate in both the FT12 and FT16 was very high at all times (>85%). After giving written informed consent, participants from both cohorts were surveyed at the baseline and the subsequent follow-ups concerning their health habits and attitudes, symptom checklists, personality scales and social relationships. Furthermore, all twins donated blood samples during a visit to the twin research clinic in Helsinki, Finland at the last follow-up (young adulthood) for genetic and biochemistry analyses. These biological samples were stored subsequently at the National Institute for Health and Welfare inside freezers at -80°C. Data collection and analysis were approved by the ethics committees of the Department of Public Health of the University of Helsinki, the Helsinki and Uusimaa Hospital District and the IRB of Indiana University. Similar to what done in TwinGene, only one individual from each twin pair was included in the present analysis.

#### Predict-CVD

The Predict-CVD sample (used here as Finnish controls) belongs to the larger FINRISK study. In brief, FINRISK is a cross-sectional population surveys originally created to investigate incident cardiovascular diseases within Finnish population by collecting information on relevant chronic diseases (e.g. CVD, diabetes, obesity, cancer) and health related behaviors in the adult population. The survey has been carried out every 5 years since 1972. DNA samples were collected in the following survey years: 1987, 1992, 1997, 2002, 2007, and 2012. A more detailed description of the FINRISK study can be found elsewhere [22]. 

The Predict-CVD sub-cohort is a random subset of the whole FINRISK cohort, and as such, representative of the full study population. The participants of the Predict-CVD were selected using random sampling stratified by sex and cohort (i.e. FINRISK 1992, 1997, 2002 or 2007 cohorts), so that each sub-cohort member had a sex/cohort specific equal sampling weight [23]. The size of sub-cohort in each stratum was made proportional to the number of incident cardiovascular disease cases in the corresponding stratum.

### Genotyping

TwinGene and the Swedish schizophrenia study were genotyped with Illumina HumanOmniExpress (≈730,000 SNPs). TwinGene genotyping was performed at the Uppsala University SNP Technology Platform (www.genotyping.se). Schizophrenia control samples were genotyped by the Genetic Analysis Platform at The Broad Institute of Harvard and MIT. Both facilities followed standard protocols and use same genotype calling (GenomeStudio). All Finnish twins and controls were genotyped using the Illumina 670K custom chip at the Welcome Trust Sanger Centre. 

### Statistical Analysis

TwinGene and Schizophrenia controls used study-specific quality control criteria. In order to harmonize the data we performed an additional quality control after merging the genotype data from the two studies. Specifically, SNPs which were not present in both studies were removed, SNPs with call rate <97%, minor allele frequency <1% or Hardy Weinberg equilibrium exact test P-value < 1x10^-7^ were also removed. Moreover, we excluded individuals with missing genotype data > 3% and we corrected for deviating heterozygosity excluding individuals with an F inbreeding coefficient calculated using PLINK [24] larger than 5 standard deviations from the sample mean. To account for population stratification we adjusted our analysis for 3 multidimensional scaling coefficients (MSCs). 

More than 100,000 SNPs were removed because of SNP call-rate < 97%. This is expected when two non-imputed datasets, which have already been processed with separate quality controls, are combined. In particular, all those SNPs that were present in only one of the two studies were excluded. After quality control, 572,352 SNPs and 10,584 individuals (6,864 twins and 3,720 controls) were included in stage 1 analysis. For each SNP we performed an additive logistic model for association with being a twin (MZ and DZ together or DZ separately) adjusting for birth year, sex and MSC. 

We performed *in silico* replication analysis in 3,372 Finnish individuals (1,492 twins and 1,880 controls) for SNPs showing association with being a twin in stage 1 analysis at P-value < 1 × 10^-5^. Replications samples followed similar quality control and merging procedures used in the Swedish samples. Logistic regression adjusting for birth year, sex and MSC was used to estimate the association between the SNPs selected in stage 1 and being a twin. Results form stage 1 and 2 were meta-analyzed using a random effects model based on inverse-variance-weighting. 

Population stratification was monitored by inspecting the three first 3 MSCs in the Finnish and Swedish sample (Figure **S1**). Lack of population stratification is further supported by the low inflation factor λ=1.03 observed in the GWAS performed in stage 1.

To perform power calculations we used the CaTs calculator [25] with a genome-wide significance P-value threshold of 5 × 10^-8^. 

We estimated the proportion of variance explained by common SNPs in our genotyping array using the Genome-wide Complex Trait Analysis (GCTA) software [26]. It has been shown that a stringent quality control is needed to increase the quality of the estimations [27] and therefore we have restricted the analysis on SNPs with minor allele frequency > 0.05 and excluded individuals with a pairwise pedigree relationship > 0.025. To transform the estimate of the heritability from the observed to the liability scale we assumed a prevalence of DZ and MZ of 2.8% and 1.1%, respectively. 

## Results

Main characteristics of our populations are described in Table **1**. In the Swedish samples, twins were older than controls but with similar sex distribution; approximately 80% of the twins were DZ. In the Finnish samples twins were in average 22 years younger than controls. In Table **2** we report the loci associated with being a twin (MZ+DZ) at P-value < 1 × 10^-5^ in stage 1. No SNP displayed genome-wide significant association (P-value < 5 × 10^-8^) with being a twin. The lowest P-values were observed for rs2033541 close to *ADAMTSL1*, rs1554783 close to *SYNE1* and rs4149283 close to ABCA1, None of these SNPs showed genome-wide significant association in meta-analysis with the Finnish samples (Table **2**).

**Table 1:** Baseline Characteristics.

		**Stage 1**		**Stage 2 - in silico replication**	
		**TwinGene - Cases (N=6864)**	**ISC - Controls (N=3720)**	**Finnish Twins - Cases (N=1492)**	**PreCVD - Controls (N=1880)**
**Sex - No. (%)**					
	**Male**	3275 (48)	1919 (52)	841 (56)	1189 (63)
	**Female**	3589 (52)	1801 (48)	653 (44)	691 (37)
**Average birth year - yr. (sd)**		1941 (8.9)	1952 (10.9)	1967 (18)	1945 (13)
**Zygosity - No (%)**					
	**Monozygotic**	1413 (21)	-	463 (31)	-
	**Dizygotic**	5451 (79)	-	1029 (69)	-

**Table 2:** Results for Association with twinness (MZ+DZ) for SNPs with P-value < 1 × 10^-5^ in Stage 1.

**Chromosome**	**SNP**	**Position (build 36)**	**Closest Gene**	**Risk Allele**	**Stage 1 - Swedish samples**			**Stage 2 - Finnish samples**			**Meta-analysis - stage 1 + stage 2**	
					**Risk allele frequency**	**OR (95% CI)**	**P-value**	**Risk allele frequency**	**OR (95% CI)**	**P-value**	**OR (95% CI)**	**P-value**
6	rs1554783	152877077	SYNE1	G	0.25	1.22 (1.13-1.32)	3.26E-07	0.28	0.98 (0.85-1.12)	0.763	1.10 (0.89-1.37)	3.78E-01
7	rs10268314	87169669	ABCB1	C	0.11	0.79 (0.71-0.87)	4.39E-06	0.06	0.90 (0.70-1.16)	0.414	0.80 (0.73-0.89)	8.56E-06
7	rs6961624	95037661	PON2	C	0.28	0.85 (0.79-0.91)	7.18E-06	0.43	0.96 (0.84- 1.09)	0.496	0.89 (0.79-1.00)	4.89E-02
9	rs2033541	18789352	ADAMTSL1	A	0.28	0.83 (0.77-0.89)	1.64E-07	0.23	0.90 (0.77-1.04)	0.145	0.84 (0.79-0.90)	1.67E-07
9	rs4149283	107626189	ABCA1	T	0.03	0.63 (0.52-0.75)	5.80E-07	0.04	0.89 (0.63-1.25)	0.503	0.73 (0.52-1.01)	5.92E-02
9	rs7035023	133797557	FIBCD1	C	0.21	0.83 (0.77-0.90)	2.27E-06	0.21	1.01 (0.87-1.17)	0.918	0.90 (0.75-1.09)	3.01E-01
10	rs11593057	14887979	HSPA14	T	0.15	0.81 (0.74-0.88)	3.31E-06	0.14	0.95 (0.80-1.14)	0.602	0.86 (0.74-1.01)	6.02E-02
10	rs564281	29441347	-	T	0.44	1.16 (1.09-1.24)	9.41E-06	0.48	1.052 (0.93-1.19)	0.431	1.12 (1.02-1.23)	1.44E-02
10	rs7081193	128074225	ADAM12	C	0.07	0.75 (0.66-0.85)	8.04E-06	0.07	1.172 (0.93-1.48)	0.182	0.93 (0.60-1.43)	7.33E-01

In-*silico* replication in stage 2 and meta-analysis of the two stages.

OR and P-values are obtained from logistic regressions adjusted for birth year, sex and 3 MSC’s or from random effect meta-analysis

We performed a secondary GWA analysis with the same set of controls but this time including only DZ twins as cases. Results are shown in Table **3**. When DZ twins were considered alone, rs2033541 close to *ADAMTSL1* and rs4149283 close to ABCA1 reached genome-wide significance (Figures **S2** and **S3**). The same direction of association was observed in the Finnish samples, albeit the association was not significant at a nominal P-value of 0.05. However, when meta-analyzed, the two SNPs reached genome-wide significant association (rs2033541: OR=0.84, P-value=5 × 10^-9^; rs4149283: OR=0.64, P-value=4 × 10^-8^).

**Table 3:** Results for Association with Dizygous (DZ) twinness for SNPs with P-value < 1 × 10^-5^ in Stage 1.

**Chromosome**	**SNP**	**Position (build 36)**	**Closest Gene**	**Risk Allele**	**Stage 1 - Swedish samples**			**Stage 2 - Finnish samples**			**Meta-analysis - stage 1 + stage 2**	
					**Risk allele frequency**	**OR (95% CI)**	**P-value**	**Risk allele frequency**	**OR (95% CI)**	**P-value**	**OR (95% CI)**	**P-value**
2	rs10928585	137768012	THSD7B	A	0.07	0.73 (0.65-0.82)	1.96E-07	0.06	0.83 (0.63-1.08)	1.71E-01	0.74 (0.67-0.83)	8.04E-08
2	rs2961957	155618326	KCNJ3	G	0.52	0.85 (0.78-0.92)	9.45E-06	0.51	1.00 (0.88-1.15)	9.71E-01	0.91 (0.78-1.07)	2.62E-01
3	rs936839	127498978	MGLL	T	0.08	0.77 (0.69-0.86)	3.60E-06	0.09	1.10 (0.86-1.40)	4.43E-01	0.90 (0.64-1.28)	5.72E-01
5	rs260409	1887817	-	T	0.19	1.19 (1.11-1.29)	6.49E-06	0.19	0.83 (0.69-0.99)	4.00E-02	1.00 (0.70-1.43)	9.95E-01
6	rs1554783	152877077	SYNE1	G	0.25	1.21 (1.12-1.29)	1.78E-07	0.28	0.94 (0.81-1.10)	4.40E-01	1.08 (0.84-1.38)	5.47E-01
7	rs2237562	86422232	GRM3	C	0.28	0.86 (0.81-0.92)	5.38E-06	0.31	1.01 (0.87-1.16)	9.38E-01	0.92 (0.79-1.07)	2.62E-01
7	rs10268314	87169669	ABCB1	C	0.11	0.79 (0.72-0.87)	1.21E-06	0.06	0.93 (0.70-1.23)	5.83E-01	0.81 (0.72-0.91)	3.73E-04
**9**	**rs2033541**	**18789352**	**ADAMTSL1**	**A**	**0.29**	**0.83 (0.78-0.88)**	**1.41E-08**	**0.23**	**0.87 (0.74-1.02)**	**7.80E-02**	**0.84 (0.79-0.89)**	**5.37E-09**
**9**	**rs4149283**	**107626189**	**ABCA1**	**T**	**0.03**	**0.62 (0.52-0.74)**	**4.30E-08**	**0.04**	**0.77 (0.52-1.11)**	**1.69E-01**	**0.64 (0.55-0.75)**	**3.78E-08**
9	rs7035023	133797557	FIBCD1	C	0.21	0.85 (0.79-0.91)	8.02E-06	0.21	1.00 (0.85-1.17)	9.65E-01	0.90 (0.78-1.05)	1.96E-01
10	rs7905434	1567107	ADARB2	G	0.45	0.87 (0.82-0.93)	7.58E-06	0.39	0.97 (0.84-1.11)	6.03E-01	0.90 (0.82-0.99)	2.68E-02
10	rs11593057	14887979	HSPA14	T	0.15	0.82 (0.76-0.90)	4.01E-06	0.14	0.99 (0.83-1.20)	9.06E-01	0.88 (0.74-1.05)	1.65E-01
10	rs564281	29441347	-	T	0.44	1.17 (1.10-1.24)	4.88E-07	0.48	1.00 (0.88-1.15)	9.80E-01	1.10 (0.94-1.27)	2.31E-01
10	rs534371	29441360	-	G	0.49	1.16 (1.09-1.23)	1.57E-06	0.52	0.98 (0.86-1.12)	7.64E-01	1.08 (0.91-1.27)	3.73E-01
10	rs7081193	128074225	ADAM12	C	0.07	0.77 (0.68-0.86)	4.91E-06	0.07	1.17 (0.91-1.50)	2.16E-01	0.94 (0.62-1.41)	7.48E-01
12	rs7955592	29778035	TMTC1	A	0.26	1.18 (1.10-1.26)	5.34E-06	0.26	0.99 (0.85-1.15)	8.42E-01	1.09 (0.92-1.30)	3.23E-01
15	rs3784415	88653481	NTRK3	C	0.10	0.8 (0.73-0.88)	7.32E-06	0.07	1.11 (0.85-1.44)	4.38E-01	0.92 (0.67-1.26)	6.07E-01

In-*silico* replication in stage 2 and meta-analysis of the two stages.

OR and P-values are obtained from logistic regressions adjusted for birth year, sex and 3 MSC’s or from random-effect meta-analysis

No genome-wide significant associations were observed when MZ were considered alone, neither in stage 1 nor in meta-analysis (Table **4**)**.**

**Table 4:** Results for Association with Monozygous (MZ) twinness for SNPs with P-value < 1 × 10^-5^ in Stage 1.

**Chromosome**	**SNP**	**Position (build 36)**	**Closest Gene**	**Risk Allele**	**Stage 1 - Swedish samples**			**Stage 2 - Finnish samples**			**Meta-analysis - stage 1 + stage 2**	
					**Risk allele frequency**	**OR (95% CI)**	**P-value**	**Risk allele frequency**	**OR (95% CI)**	**P-value**	**OR (95% CI)**	**P-value**
2	rs11891511	240644907	-	G	0.18	1.28 (1.15-1.43)	7.66E-06	0.18	0.91 (0.70-1.78)	0.474	1.10 (0.79-1.54)	5.68E-01
7	rs10487484	127105414	-	C	0.11	1.36 (1.19-1.55)	9.10E-06	0.16	1.04 (0.80-1.36)	0.754	1.22 (0.95-1.57)	1.25E-01
14	rs1152790	99710843	BCL11B	T	0.50	1.18 (1.11-1.25)	6.77E-06	0.49	1.10 (0.90-1.34)	0.363	1.17 (1.08-1.26)	1.75E-04
15	rs8029801	102073917	-	T	0.18	1.30 (1.17-1.45)	2.31E-06	0.19	0.73 (0.56-0.95)	0.02	0.99 (0.56-1.74)	9.61E-01
17	rs3826503	3474660	TRPV1	A	0.18	0.74 (0.66-0.83)	8.79E-07	0.24	0.95 (0.75-1.20)	0.688	0.82 (0.65-1.05)	1.18E-01
18	rs4552096	74518951	-	T	0.43	0.81 (0.74-0.89)	5.68E-06	0.41	1.13 (0.92-1.39)	0.233	0.95 (0.68-1.31)	7.41E-01

In-*silico* replication in stage 2 and meta-analysis of the two stages.

OR and P-values are obtained from logistic regressions adjusted for birth year, sex and 3 MSC’s or from random-effect meta-analysis

To investigate what effect-size we had power to detect in the current setting we performed a power calculation [25]. The prevalence of twins in the investigated age-group is close to 2%. With such prevalence and assumption of allele frequency of 0.5 we had, in the stage 1 analysis, 76% and 95% power to detect odds ratios of 1.21 and 1.25, respectively. The same OR would have been detected with a power of 48% and 80% considering an allele frequency of 0.2. 

We estimated that the proportion of phenotypic variance on liability scale explained by all common SNPs available in our genotyping array (“chip heritability”) was 0.06 [standard error (s.e.): 0.05, P-value=0.10] in MZ and 0.09 (s.e: 0.03, P-value=0.003) in DZ twins. When MZ and DZ twins were analyzed together, the estimated proportion was 0.08 (s.e: 0.03, P-value=0.003).

## Discussion

We conducted this analysis to test the hypothesis that twins have similar genetic architecture as singletons. If this is the case, biological twinness does not confound GWAS, and as consequence, genetic data derived from twins can be used in such studies without introducing large bias. On the other hand, if genetic variants associated with biological twinness exist, they can bias GWAS in two ways. First, if the twins are used as healthy controls in disease-specific GWAS, spurious association can be detected. Second, if the GWAS is conducted only on twins or in combined samples where twins and non-twins are analyzed together (for example to investigate a continuous phenotype) spurious association can be detected if the genetic variants associated with biological twinness are also associated with the phenotype of interest.

In the present study we did not find common genetic variants that were associated with being a twin. In a secondary analysis of DZ twins versus non-twins, we identified two SNPs that were genome-wide significant in the stage 1 and when meta-analysed with the Finnish samples. Given our sample size, we have 80% power to detect genetic variants with an effect size of 1.22 and 1.25 and an allele frequency of 0.50 and 0.20, respectively. Therefore, rarer variants or variants with smaller effect size might have been missed in this study. To evaluate the potential for discovery of SNPs associated with twinness, we estimated the proportion of variance on liability scale explained by all common SNPs available in our genotyping array. A high proportion of variance would indicate that common SNPs play a large role and thus, that many associated SNPs might be discovered with larger samples. We observed a small proportion of variance explained (0.06 for MZ and 0.09 for DZ twins) indicating that there is, relatively speaking, a limited potential for discovery of many genetic variants associated with biological twinness 

Twins samples have already been used in several GWA studies, together with other singletons samples or independently. A genotyped sample of individuals from the UK twin registry has been part of a large number of GWA meta-analysis [28]. In 2010, more than 30 GWA meta-analyses have been published using these data (http://www.twinsuk.ac.uk/publications.html#2010). GWA studies have also been performed only using samples of related individuals. For example, a GWAS for association with height and body mass index has been conducted in a sub-sample of the Australian Twin Registry including 11,536 individuals composed of Australian twins, family members, and unrelated individuals [29]. The authors reported a single genome- wide significant variant for height with an effect size that was comparable with that observed in a previous GWA study of unrelated individuals [30]. Genome-wide association meta-analysis consortia conventionally perform quality controls to identify heterogeneity among studies. It might be argued that, if twins were genetically different from singletons, this would already have been picked up and reported by such consortia. This is however true only if a large part of the genetic associations in a specific study differ from those detected in the other studies (e.g. due to population stratification). Instead, it would not be reported if a few variants, for example those that hypothetically might be associated with being a twin, were heterogeneous across studies. The only exception would be when these variants also happened to be the top-findings of the disease investigated by the GWAS. This study is, to our knowledge, the first genome-wide investigation comparing genetic variants in twins and singletons from the same underlying population. An important strength is that the samples were handled and extracted by the same biobank and genotyped with identical platforms, minimizing potential biases due to technical differences between platforms and providing the advantage of not having to rely on imputation. 

Although practically identical procedures and platforms were used, the twins and singletons were genotyped in different laboratories and at different time-points. This might have introduced systematic bias by increasing the risk to detect spurious association and by inflating the estimates of the proportion of variance explained by all common SNPs. However, these are issues only if positive findings are observed. Because our main analysis on twins compared to non-twins revealed no significant locus and the estimated “chip-heritability” was low, we consider the effect of this potential bias negligible. 

It is important to highlight that the aim of this study was not to identify new genetic variants predisposing to twinning, for example, by influencing the probability of blastocyst fragmentation, multiple ovulation or mere survival of more than one concomitant fetus. We recognize that to pursue this aim a better approach would be to investigate the genetic architecture of parents of MZ and DZ twins. Other studies with larger sample size are needed to investigate these hypotheses. Nevertheless, we identified two loci, ADAMTSL1 and ABCB1, likely to be associated with DZ twinness. ABCA1 is involved in cholesterol transport and SNP markers on the same loci have been found previously associated with HDL-levels in several large GWAS [31-33]. Further research is needed to confirm these findings and elucidate the potential role of these loci in relation to factors predisposing to DZ twinning.

We used a Finnish population to replicate SNPs with a p-value < 1 × 10^-5^ in the main analysis. Finns are considered an outlier population on the European genetic landscape [34], other populations such as Danes or Germans could be argued to be more suitable to replicate findings from Swedish individuals. However, genetic data from Finnish population, including twins from the Finnish Twin Cohort, have been used in a large number of GWA studies (http://www.euengage.org/press.html) and results have been reasonably comparable to those observed in other European populations. In addition, it should be acknowledged that, while we know that both Predict-CVD study sample and the wave 5 and 6 of the Swedish schizophrenia study do not include pairs of relatives, it is uncertain whether any of the participants to these studies could be a member of a twin pair. Notwithstanding, while this may potentially introduce an error in our analyses, it may be safely neglected as twins would represent ~2% of the participants in these population-based samples. 

In conclusion, we did not find evidence for large genetic differences between twins and singletons, supporting the practice to use twins together with singletons in genetic studies without introducing bias.

## Supporting Information

Figure S1 **Plot of the first 3 MDCs from the Finnish samples (Panel A.) and Swedish samples (Panel B).**
(JPG)

Figure S2 **The plot is centered on rs2033541 (purple diamonds).**The R^2^ values are from the CEU HapMap2 samples. The CEU HapMap2 recombination rates are indicated in blue on the right y axes. The figures were created with LocusZoom (http://csg.sph.umich.edu/locuszoom/). Mb, megabases.
(PDF)

Figure S3 **The plot is centered on rs4149283 (purple diamonds).**The R^2^ values are from the CEU HapMap2 samples. The CEU HapMap2 recombination rates are indicated in blue on the right y axes. The figures were created with LocusZoom (http://csg.sph.umich.edu/locuszoom/). Mb, megabases.
(PDF)
