# Supplementary figures and images for: Utilizing Twins as Controls for Non-Twin Case-Materials in Genome Wide Association Studies
Source: PLoS One. 2013 Dec 10;8(12):e83101. doi: 10.1371/journal.pone.0083101 (PMC3858365; doi:10.1371/journal.pone.0083101)

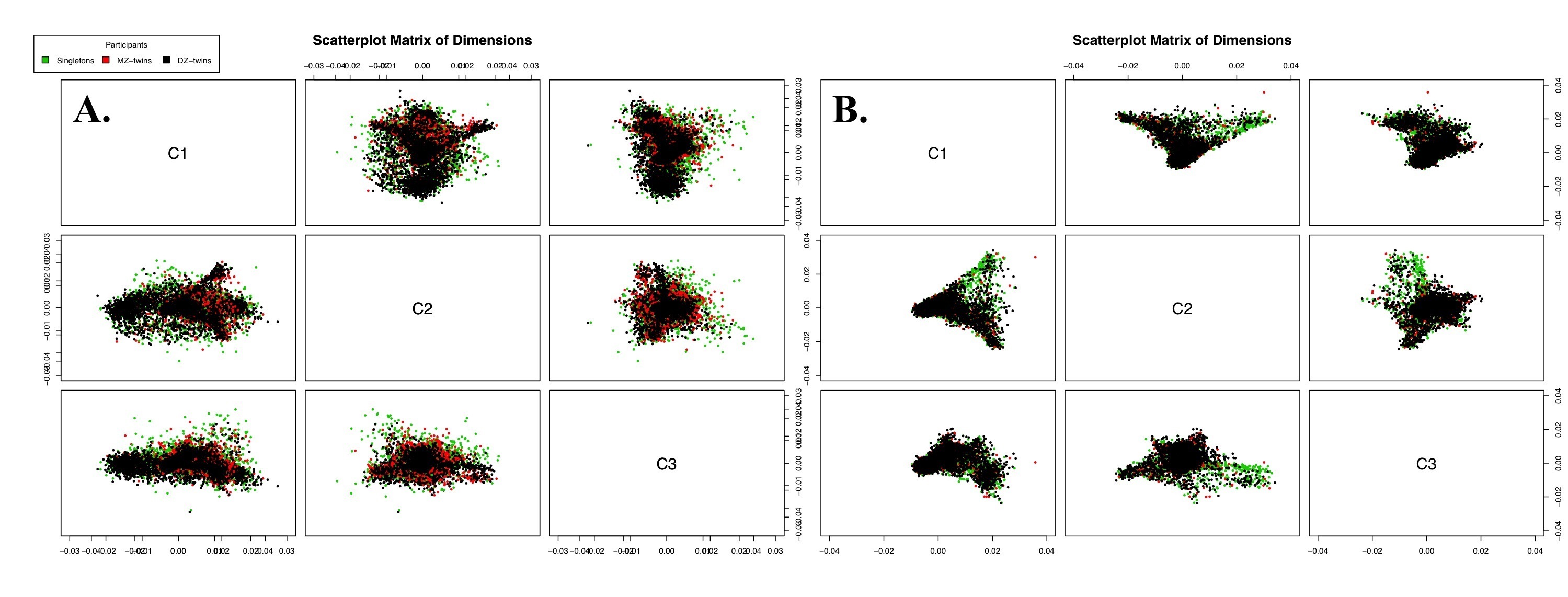

Supplement: Figure S1 — Plot of the first 3 MDCs from the Finnish samples (Panel A.) and Swedish samples (Panel B). (JPG) [file pone.0083101.s001.jpg]
