# Supplementary material for: Utilizing Twins as Controls for Non-Twin Case-Materials in Genome Wide Association Studies
Source: PLoS One. 2013 Dec 10;8(12):e83101. doi: 10.1371/journal.pone.0083101 (PMC3858365; doi:10.1371/journal.pone.0083101)
Supplement: Figure S2 — The plot is centered on rs2033541 (purple diamonds).The R2 values are from the CEU HapMap2 samples. The CEU HapMap2 recombination rates are indicated in blue on the right y axes. The figures were created with LocusZoom (http://csg.sph.umich.edu/locuszoom/). Mb, megabases. (PDF) [file pone.0083101.s002.pdf]

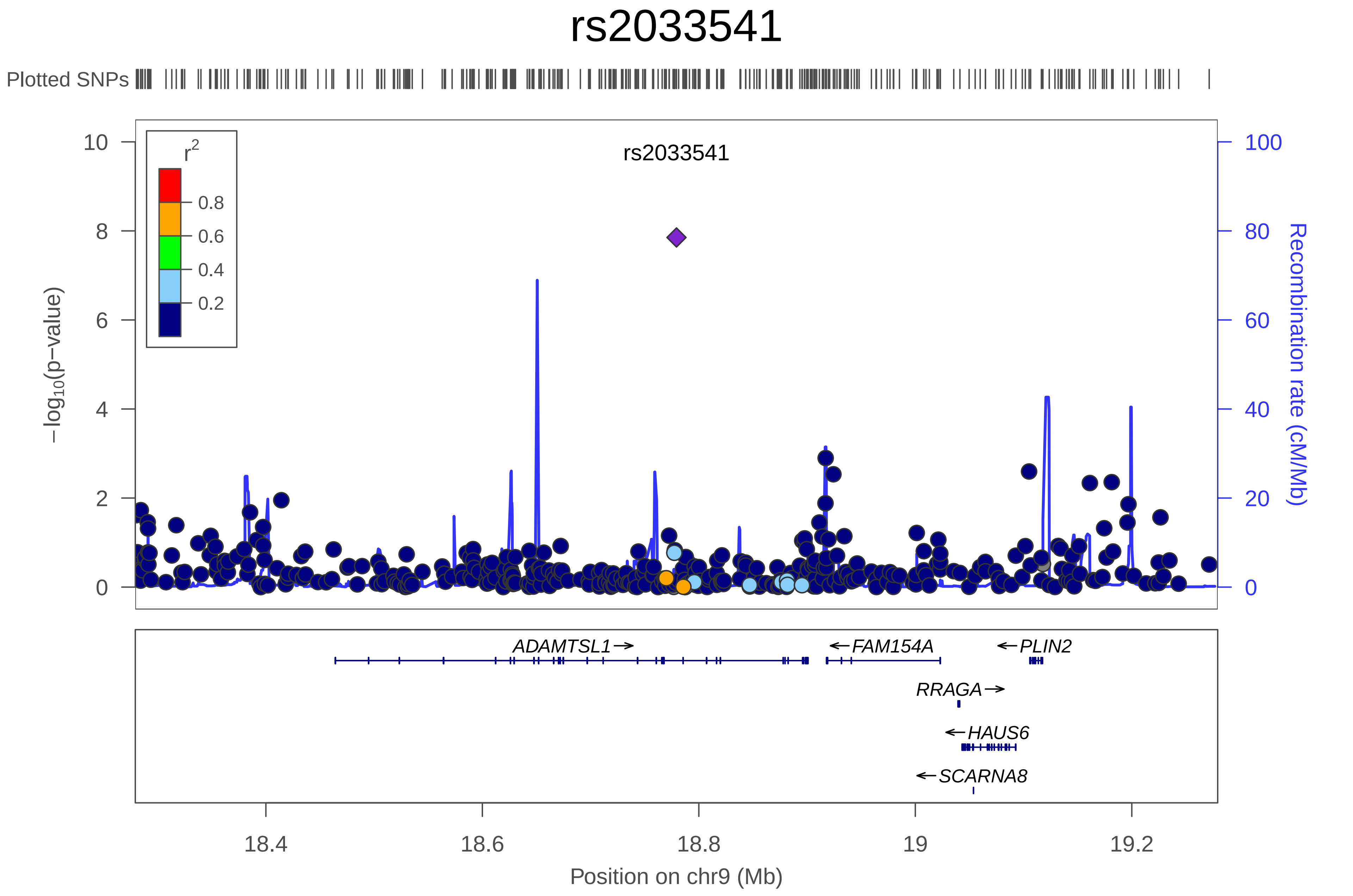

rs2033541
Plotted SNPs
r2
0.8
0.6
0.4
0.2
rs2033541
−log10(p−value)
10
8
6
4
2
0
100
80
60
40
20
0
Recombination rate (cM/Mb)
ADAMTSL1→
←FAM154A
←PLIN2
RRAGA→
←HAUS6
←SCARNA8
18.4
18.6
18.8
19
19.2
Position on chr9 (Mb)

date:	Mon Oct 14 10:30:11 2013
build:	hg18
display range:	chr9:18279352−19279352 [18279352−19279352]
hilite range:	0 − 0 [0 − 0]
reference SNP:	chr9:18779352
number of SNPs plotted:	364
max P:	1.4E−8 [chr9:18779352]
min P:	10E−1 [chr9:18787256]

Make more plots at http://csg.sph.umich.edu/locuszoom/
