# Supplementary material for: Utilizing Twins as Controls for Non-Twin Case-Materials in Genome Wide Association Studies
Source: PLoS One. 2013 Dec 10;8(12):e83101. doi: 10.1371/journal.pone.0083101 (PMC3858365; doi:10.1371/journal.pone.0083101)
Supplement: Figure S3 — The plot is centered on rs4149283 (purple diamonds).The R2 values are from the CEU HapMap2 samples. The CEU HapMap2 recombination rates are indicated in blue on the right y axes. The figures were created with LocusZoom (http://csg.sph.umich.edu/locuszoom/). Mb, megabases. (PDF) [file pone.0083101.s003.pdf]

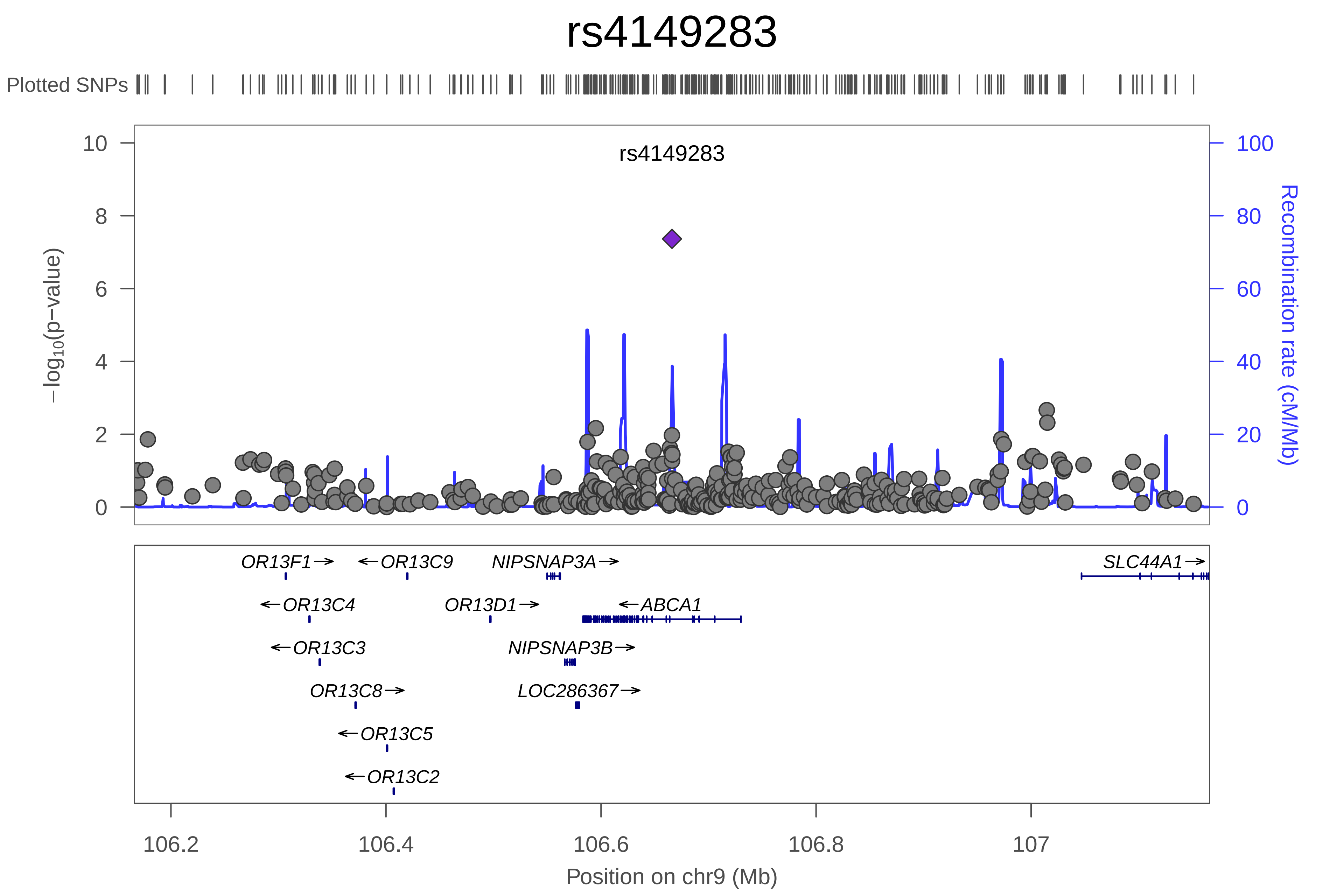
rs4149283
Plotted SNPs
rs4149283
10
8
6
4
2
0
−log10(p−value)
100
80
60
40
20
0
Recombination rate (cM/Mb)
OR13F1→
←OR13C9
NIPSNAP3A→
SLC44A1→
←OR13C4
OR13D1→
←ABCA1
←OR13C3
NIPSNAP3B→
OR13C8→
LOC286367→
←OR13C5
←OR13C2
106.2
106.4
106.6
106.8
107
Position on chr9 (Mb)

date:	Mon Oct 14 10:31:28 2013
build:	hg18
display range:	chr9:106166010−107166010 [106166010−107166010]
hilite range:	0 − 0 [0 − 0]
reference SNP:	chr9:106666010
number of SNPs plotted:	369
max P:	4.3E−8 [chr9:106666010]
min P:	9.98E−1 [chr9:106400672]
Warning:	No usable LD information for reference SNP.

Make more plots at http://csg.sph.umich.edu/locuszoom/
